# Does Experience Enhance Cognitive Flexibility? An Overview of the Evidence Provided by the Environmental Enrichment Studies

**DOI:** 10.3389/fnbeh.2019.00150

**Published:** 2019-07-09

**Authors:** Francesca Gelfo

**Affiliations:** ^1^Department of Human Sciences, Guglielmo Marconi University, Rome, Italy; ^2^Department of Clinical and Behavioural Neurology, IRCCS Fondazione Santa Lucia, Rome, Italy

**Keywords:** neuroplasticity, cerebral reserve, environmental enrichment, cognitive flexibility, animal models, rodents, reversal learning, attentional set-shifting

## Abstract

Neuroplasticity accounts for the ability of the brain to change in both structure and function in consequence of life experiences. An enhanced stimulation provided by the environment is able to create a form of brain, neural, and cognitive reserve, which allows an individual to cope better with the environmental demands, also in case of neural damage leading to cognitive decline. With its complex manipulation of several stimuli, the animal experimental paradigm of environmental enrichment (EE) appears particularly effective in modulating the ability to successfully respond to the ever-changing characteristics of the environment. According to this point, it could be very relevant to analyze the specific effects of EE on cognitive flexibility (CF). CF could be defined as the ability to effectively change behavior in response to the environmental condition changing. This review article is specifically aimed to summarize and focus on the available evidence in relation to the effects of EE on CF. To this aim, findings obtained in behavioral tasks specifically structured to investigate animal CF, such as reversal learning and attentional set-shifting tests (tasks based on the request of responding to a rewarding rule that changes, within one or multiple perceptual dimensions), are reviewed. Data provided on the structural and biochemical correlates of these findings are also enumerated. Studies realized in healthy animals and also in pathological models are considered. On the whole, the summarized evidence clearly supports the specific beneficial effects of EE on CF. However, further studies on this key topic are strictly required to gain a comprehensive and detailed framework on the mechanisms by which an enhanced stimulation could improve CF.

## Experience and Neuroplasticity

From the late 1940s, the idea that the brain has not a fixed structure but is characterized by a deep plasticity has been largely recognized (Hebb, 1949). As it is now known, every experience is translated in our brain in electrochemical messages, which allow us to feel, perceive, and elaborate information regarding each event occurring inside and outside the body. Such electrical activity induces plastic changes in the brain, both at a structural and at a functional level (Sale et al., 2014; Li et al., 2019). The ability of the brain to change in consequence of the experience is called *neuroplasticity* and is the basis of its successful coping with a number of situations, such as development, injury, and also usual learning and memory processes (Griesbach and Hovda, 2015; Gulyaeva, 2017).

Indeed, studies on cognitive decline demonstrated that such plastic changes could empower the brain with a form of “reserve.” Namely, subjects with different sets of life experiences show different rate in developing physiological cognitive decline and/or dementia, with no linear correlation with brain structural degeneration (Stern, 2002; Perneczky et al., 2019). On this basis, the concept of cerebral reserve has been developed in relation to the experience-induced brain structure and function rearrangements that are able to support high-level performance in demanding situations and tasks, both in physiological and pathological conditions, which could be linked to age-related neurodegeneration or to other kinds of brain damage. The concept of reserve comprises a number of different levels, such as: (i) the *brain reserve*, that refers to the characteristics of the brain structural asset of an individual (such as brain volume; amount and morphology of neuronal and glial cells, blood vessels, and synapses; expression levels of neurotransmitters and neuromodulators; etc.); (ii) the *neural reserve*, that refers to the efficiency of the neural circuitry of an individual; and (iii) the *cognitive reserve*, that refers to the cognitive abilities and strategies recruited in cognitive and behavioral tasks by an individual (Serra et al., 2018; Stern et al., 2018; Serra and Gelfo, 2019). The reserve in its different aspects can be evaluated in subjects both in healthy and pathological conditions, by comparing them to subjects characterized by a lower level of experience-due stimulation in the previous life period.

It is widely agreed in the literature (Clare et al., 2017; Mandolesi et al., 2017; Chan et al., 2018; Pettigrew et al., 2019) that it is possible to identify three main lifestyle factors that induce a neuroprotective effect in the brain: (i) the *social factor*, that encompasses all the activities and engagements which include an individual in a high-level social network (such as familiar status; parentage; family ties; companionship; etc.; Bennett et al., 2014; Kuiper et al., 2016; Evans et al., 2018); (ii) the *cognitive factor*, that encompasses all the mentally demanding activities in which an individual could be significantly involved (such as education; occupational attainment; leisure activities; etc.; Yates et al., 2016; Grønkjær et al., 2019; Pudas and Rönnlund, in press); and (iii) the *physical factor*, that encompasses all the habits of healthy living maintained by an individual (such as motor activity; healthy dietary routines; intake of specific beneficial dietary components, such as ω-3 polyunsaturated fatty acid or antioxidants; abstention by alcohol massive assumption and smoking etc.; Lista and Sorrentino, 2010; Christie et al., 2017; Phillips, 2017; Blanchet et al., 2018; Rossi Dare et al., 2019). An enriching life-experience in the three listed dimensions seems to act just in an opposite and independent manner in respect to stressful early-life experiences, which appear to constitute a risk factor for vulnerability to pathological cognitive decline (Cabral et al., 2016; Caruso et al., 2018). On the other hand, it is important to add that usually, in human life-experience, these factors do not act independently, but are inter-related, and the brain structural, neural and functional status of an individual is the result of a sum of influences (Perneczky et al., 2019).

Clinical studies provided the cerebral reserve theory with large evidence. Nevertheless, some limitations of these studies have been highlighted, since genetic and life-experience peculiarities make humans hardly comparable on the basis of clearly defined and controlled variables (primarily due to the inter-related action of the listed factors in everyone life-experience). Moreover, a number of structural and biochemical aspects of the reserve are hardly evaluable in human *in vivo* studies (Petrosini et al., 2009; Gelfo et al., 2018; Serra et al., 2018; Serra and Gelfo, 2019). Such limitations could be overcome by modeling in animals the effects of the experience reported in humans, with the use of the environmental enrichment (EE) experimental paradigm.

## The Environmental Enrichment

The EE paradigm was introduced in the 1960s, to test in animals the effects of an enhanced social, cognitive and physical stimulation (Diamond et al., 1966; Rosenzweig and Bennett, 1996). The EE apparatus is frequently used with rodents, in comparison to the standard laboratory housing conditions (two/four animals/cage; standard cage size and bedding; *ad libitum* food and water; Nithianantharajah and Hannan, 2006, 2009). In this apparatus, by manipulating selected and well-defined variables for determined time-periods, it is possible to mimic the range of human experiences in relation to the three lifestyle factors: (i) the *social factor* is mimicked by housing the animals in groups larger than the usual ones; (ii) the *cognitive factor* is mimicked by housing the animals in complex environments, containing a lot of objects that are repeatedly substituted and repositioned, such introducing continuous elements of novelty; and (iii) the *physical factor* is mimicked by housing the animals in large cages that stimulate the exploratory movements, providing the cages with ladders and running wheels that allow motor activity, and also by offering to the animals specific supplementary diet elements (Baroncelli et al., 2010; Mo et al., 2016; Sampedro-Piquero and Begega, 2017). The EE paradigm represents a versatile mean to test the effects of all the involved factors or only one of them, by determining the starting, the end, and the duration of the exposure, and also by choosing the sensory channel to be mainly stimulated. Further, it is possible to determine also if exposing to EE animals in healthy or pathological conditions (Simpson and Kelly, 2011; Toth et al., 2011; Dolivo and Taborsky, 2017; Gelfo et al., 2018).

On the whole, animal studies that investigated the effects of the exposure to EE stably reported an improvement in cognitive and behavioral performance, both in healthy and pathological conditions leading to cognitive decline (Leggio et al., 2005; Nithianantharajah and Hannan, 2006, 2009; Petrosini et al., 2009; Foti et al., 2011; Hannan, 2014; Mandolesi et al., 2017). These results are accompanied by a strengthening of brain structure, neural circuitry and neurobiological processes (Kondo, 2017; Gelfo et al., 2018). More specifically, EE is reported to increase neurogenesis (Bergami, 2015; Sakalem et al., 2017; Kempermann, 2019), gliogenesis (Chakrabarti et al., 2011; Freund et al., 2013), angiogenesis (He et al., 2017), and synaptogenesis (Gelfo et al., 2009, 2016; Hirase and Shinohara, 2014). Also, a number of enhancing EE effects are described in relation to molecular processes, such as neurotrophic factor expression (Gelfo et al., 2011; Mosaferi et al., 2015; Novkovic et al., 2015) and neurotransmitter system functioning (Aumann, 2016; Gonçalves et al., 2018). With the support of an improved neural structure and functionality, the enriched animals show enhanced capacity to efficiently respond to the challenging situations proposed in the behavioral tasks, by utilizing high-level strategies in the performance. These enhanced capabilities have been largely investigated in learning and memory tasks (Pang and Hannan, 2013; Jin et al., 2017; Cortese et al., 2018; Bleimeister et al., 2019). However, since a relevant effect of EE is the augmented capability of adapting to and facing the ever-changing characteristics of the environment, an intriguing issue to be investigated could regard specifically the EE effects on cognitive flexibility (CF).

## The Cognitive Flexibility

The ability to maintain in memory the representations of the information previously encountered in everyone’s experience is fundamental to successfully respond to the environmental stimuli; however, also the ability to efficiently update the retained information in consequence of the rapid changes of the environment is required for a successful adaptation (Bizon et al., 2012). CF could be defined just as the ability to shift associations and attentional sets in order to respond properly to the changing environmental conditions and demands; namely, CF is the ability to change behavior when environmental conditions change (Brigman et al., 2012; Nilsson et al., 2015). This fundamental process is encompassed among the *executive functions* (that also include working memory and inhibition), which make the individuals able to control adaptively their own thought and action (Buttelmann and Karbach, 2017). In humans, the gold standard to assess CF is the Wisconsin Card Sorting Test (Berg, 1948), which is structured to evaluate the ability to acquire association rules and attentional sets and to switch between them (Lange et al., 2017). In this test, after the acquisition of a card-sorting rule, the rule is changed, and the subject has to adapt the response to this shift. In rodents, CF is usually assessed by reversal learning or attentional set-shifting tasks. Typically, in both kinds of task a rule of reward has to be learned by the animal, and then the rule is changed, and the animal has to respond to the new rule to gain the reward (Nilsson et al., 2015; Girotti et al., 2018). In the *reversal learning* tasks, the rule associates a single discriminatory stimulus—between two in the same perceptual dimension—with a reward; when the animal learns the rule, it is reversed (Talpos and Shoaib, 2015). Differently, the *attentional set-shifting* tasks usually involve more than one perceptual dimension, each containing more than one stimulus. In a first phase, the rewarded stimulus falls in a perceptual dimension; an *intradimensional shift* may be proposed, by changing the discrimination problem, but by rewarding always the same perceptual dimension. After the acquisition of the rewarding rule, a previously irrelevant perceptual dimension becomes relevant, and the rewarded stimulus is included in this other one (*extradimensional shift*, Chudasama, 2011; Tait et al., 2014; Heisler et al., 2015). On these two basic aspects, also more complex settings to assess CF have been developed, in which the rule to be acquired may imply the choice among or the learning of a sequence of potentially rewarded stimuli (Dalley et al., 2004; De Bartolo et al., 2009; Izquierdo et al., 2017).

Classically, CF has been associated with the functioning of prefrontal cortex (Kesner and Churchwell, 2011). Specifically, it has been showed in rodents that orbitofrontal cortex seems to support the performance in reversal learning tasks, whereas the regions of medial prefrontal cortex seem to be involved in attentional set-shifting tasks (Dalley et al., 2004; Brockett et al., 2015; Izquierdo et al., 2017). However, recent studies demonstrated that a larger circuitry is involved in the efficient attentional shifting; it has been advanced that it comprises also connections with the striatum and the amygdala (Klanker et al., 2013; Izquierdo et al., 2017). Also, several studies have evidenced that the cerebellum exerts a main role in modulating prefrontal cortex functioning in CF (De Bartolo et al., 2009; Dickson et al., 2010, 2017; Shipman and Green, in press). In addition, a key-function in CF has been devoted to new neurons that integrate themselves in hippocampal circuitry by neurogenesis (Anacker and Hen, 2017). A number of neurochemical factors have been indicated to modulate CF (Izquierdo et al., 2017; Girotti et al., 2018), which seems to involve different neurotransmitter systems, such as the cholinergic (Prado et al., 2017), dopaminergic (Izquierdo et al., 2006, 2010), noradrenergic (Logue and Gould, 2014), serotoninergic (Brigman et al., 2010), and glutamatergic (Jett et al., 2017) ones.

Impairments in CF are often characteristic of physiological and pathological aging, and such deficits are reported in several species (Bizon et al., 2012; Pfeffer et al., 2018). Moreover, deficits in CF characterize a number of neuropathological conditions, such as states of inflammation (Jurgens and Johnson, 2012), neurodevelopmental disorders (Whitehouse et al., 2017), schizophrenia (Saland and Rodefer, 2011), and Huntington’s disease (Harrison et al., 2013; Curtin et al., 2016). According to this issue and to the fact that EE seems particularly suited to modulate the ability to successfully respond to the changing demands of the environment, it is noteworthy to specifically consider the EE effects on CF.

## Effects of Environmental Enrichment on Cognitive Flexibility

Given the large amount of experiments aimed to evaluate the EE effects on cognitive functions, a quite limited number of studies have been specifically devoted to investigate the EE effects on CF. By searching in PubMed for the references specifically related to EE and CF, without language and time-range limitations, and also by checking the references included in the relevant publications, the current state of the art appears to provide only 12 studies specifically devoted to the investigation of this topic.

As for the evidence in healthy animals, it has been demonstrated that a long-duration exposure to EE is able to improve the performance in tasks assessing CF, both in reversal learning and in intradimensional shift (Schrijver et al., 2004; Zeleznikow-Johnston et al., 2017; Rountree-Harrison et al., 2018). More specifically, Schrijver et al. (2004) reported that rats reared in EE (consisting in social enhanced stimulation) from weaning onwards showed in adulthood better performance in reversal learning compared to controls. This finding was not revealed after the exposure to EE consisting in inanimate stimulation. Differently, Zeleznikow-Johnston et al. (2017) reported that mice reared in EE (complex paradigm) from 4 weeks of age onwards showed in adulthood better performance in reversal learning in comparison to controls, also when social stimulation was not manipulated. Rountree-Harrison et al. (2018) confirmed that mice reared in EE (complex paradigm) from birth onwards showed in adulthood better performance in comparison to controls when tested in reversal learning, and also in intradimensional shift. Moreover, it has been demonstrated that also a brief-duration exposure to EE (complex paradigm) in adulthood is able to enhance CF performance (Brockett et al., 2015; Sampedro-Piquero et al., 2015). Sampedro-Piquero et al. (2015) showed that rats exposed to EE (complex paradigm) for 21 days in adulthood showed better performance in reversal learning; the combination of EE with a paradigm of forced exercise did not provide an additive effect. However, Brockett et al. (2015) found that rats exposed only to physical EE consisting in free running in wheels for 12 days in adulthood showed enhanced performance in reversal learning, and also in extradimensional shift. Functional effects of the exposure to EE were accompanied by enhanced activation, synaptogenesis and gliogenesis in hippocampus, perirhinal cortex, medial prefrontal cortex, and orbitofrontal cortex (Brockett et al., 2015; Sampedro-Piquero et al., 2015). Details on all of the studies cited in this section are provided in Table 1.

**Table 1:** Studies referred to the environmental enrichment effects on cognitive flexibility in healthy animals.

Reference	Species (age or weight at the start of the environmental enrichment) *Environmental enrichment type and duration*	Task assessing cognitive flexibility	Environmental enrichment effects
Brockett et al. (2015)	Male Sprague-Dawley rats (adult) *Free access to running wheel; 12 days*	Attentional set-shifting task (including: reversal learning; intradimensional shift; extradimensional shift)	Enhanced performance in reversal learning and extradimensional shift. Enhanced synaptogenesis (hippocampus; medial prefrontal cortex; orbitofrontal cortex; perirhinal cortex) and gliogenesis (hippocampus; medial prefrontal cortex; orbitofrontal cortex)
Rountree-Harrison et al. (2018)	C57Bl6 mice (from birth) *Environmental enrichment—with running wheels; 74–93 days*	Olfactory temporal order discrimination task (including: reversal learning; intradimensional shift)	Enhanced performance in reversal learning and intradimensional shift
Sampedro-Piquero et al. (2015)	Male Wistar rats (3.5 months) *Environmental enrichment: with vs. without forced exercise in Rotarod apparatus—no running wheels; 21 days*	4-Radial arm water maze task (including: reversal learning; intradimensional shift)	Enhanced performance in reversal learning, without addictive effect of forced exercise. Enhanced activation (c-Fos expression) in cingulate area of medial prefrontal cortex and orbitofrontal cortex
Schrijver et al. (2004)	Male Lister hooded rats (21 days) *Environmental enrichment: social vs. inanimate stimulations—no running wheels; 100 days*	Two-choice discrimination task (including: reversal learning)	Enhanced performance in reversal learning (social stimulation). No effect in reversal learning (inanimate stimulation)
Zeleznikow-Johnston et al. (2017)	Male wild-type C57Bl/6J mice (4 weeks) *Environmental enrichment: no social manipulation—no running wheels; 10 weeks*	Two-choice visual discrimination task (including: reversal learning)	Enhanced performance in reversal learning

*Note: the characterization reported for the environmental enrichment paradigm specifies the variables manipulated, when variations on the classical complex paradigm (described in the article) are involved*.

As for animal models mimicking aspects of human pathological conditions that provoke impairments in CF, evidence has been provided that EE exerts specific beneficial effects in tasks investigating this cognitive domain.

Specifically, it has been demonstrated that a long-term exposure (about 3 to 5 months) to EE (complex paradigm) is able to restore the performance in reversal learning both in transgenic (Pfeffer et al., 2018) and immunotoxic (De Bartolo et al., 2008) rodent models of Alzheimer’s disease. In association, an augment in hippocampal neurogenesis and gliogenesis was reported (Pfeffer et al., 2018). A similar functional result has been shown in consequence of long-term cognitive or physical enrichment (about 15–22 weeks) in mouse models of Huntington’s disease, with a reduction of the characteristic striatal neural loss (Harrison et al., 2013; Curtin et al., 2016). A 6-week exposure to EE without social manipulation (starting at weaning) has been demonstrated to enhance reversal learning also in a mouse model of neurodevelopmental disorder (Whitehouse et al., 2017). Moreover, in a rat model of schizophrenia, the exposure to physical enrichment only for 1 h a day for 30 days has been reported to improve performance both in reversal learning and extradimensional shift (Saland and Rodefer, 2011). Differently, in an adult mouse model of neuroinflammation (7-day inoculation of influenza virus), a previous 4-month exposure to EE (complex paradigm) failed in improve the performance in reversal learning, despite a reduction in hippocampal proinflammatory cytokines was reported (Jurgens and Johnson, 2012). Details on all of the studies cited in this section are provided in Table 2.

**Table 2:** Studies referred to the environmental enrichment effects on cognitive flexibility in pathological models.

Reference	Species (age or weight at the start of the environmental enrichment) *Environmental enrichment type and duration*	Pathological condition modeled	Task assessing cognitive flexibility	Environmental enrichment effect
Curtin et al. (2016)	Male and female zQ175 mice (7–8 weeks) *Early cognitive training; evaluation in adulthood*	Huntington’s disease	Two-choice visual discrimination task (including: reversal learning)	Enhanced performance in reversal learning
De Bartolo et al. (2008)	Male Wistar rats (21 days) *Environmental enrichment—no running wheels; 5 months*	Alzheimer’s disease (cholinergic immunotoxic depletion in the basal forebrain at 90 days)	Serial learning task (including: reversal learning)	Enhanced performance in reversal learning
Harrison et al. (2013)	Male R6/1 mice (5 weeks) *Access to running wheel (14 h/day; 5 days/week); 9/22 weeks*	Huntington’s disease	Water T-maze set-shifting task (including: reversal learning)	Enhanced performance in reversal learning only after 22-week exposure. Reduction of striatal neuronal loss
Jurgens and Johnson (2012)	Male BALB/c mice (7 weeks) *Environmental enrichment—with running wheels; 4 months*	Influenza infection (inoculation with influenza A7PR8/34 virus at 6 months)	Morris water maze task - day 7 post-inoculation (including: reversal learning)	No effect in reversal learning. Reduction in hippocampal inflammation (proinflammatory cytokine expression)
Pfeffer et al. (2018)	Female APP23 mice (5 weeks) *Environmental enrichment—no running wheels; 1/12/24 weeks*	Alzheimer’s disease	Morris water maze task (including: reversal learning)	Enhanced performance in reversal learning for young adults (12 weeks of exposure to EE). No effects after 1 or 24 weeks of exposure. Enhanced hippocampal neurogenesis in adolescent and young adult APP23 mice. Enhanced hippocampal gliogenesis in adult APP23 mice
Saland and Rodefer (2011)	Male Long Evans rats (51 days) *Environmental enrichment: free access to running wheels + enriched diet (alternation among water; saccharin solution; cookie); 1 h/day; 30 days*	Schizophrenia (phencyclidine HCl administration for 7 days starting at 66 days)	Two-choice discrimination task (including: reversal learning; intradimensional shift; extradimensional shift)	Enhanced performance in reversal learning and extradimensional shift
Whitehouse et al. (2017)	Female C58 mice (21 days) *Environmental enrichment: no social manipulation—with running wheels; 6 weeks*	Neurodevelopmental disorders (restricted and repetitive behavior)	Positional discrimination task (including: reversal learning)	Enhanced performance in reversal learning

*Note: the characterization reported for the environmental enrichment paradigm specifies the variables manipulated, when variations on the classical complex paradigm (described in the article) are involved. Findings reported refer to enriched animals in comparison to non-enriched animals in the same pathological condition*.

## Conclusions

To the best of my knowledge, this is the first review of the scientific literature specifically aimed to summarize the evidence related to the effects of EE on the brain structure and function supporting CF. This one appears as a core issue since EE manipulation is particularly suited to modulate brain ability to cope with the changing demands of the environment. On the whole, this review article indicates that the exposure to EE is able to exert specific beneficial effects on CF performance when assessed both in reversal learning and in attentional set-shifting tasks. This evidence is confirmed both in healthy rodents and in pathological models.

However, a number of issues remain unclear, since the number of available studies is quite limited, and the applied experimental designs are very different in relation to several fundamental variables (such as age of EE starting; duration of the exposure to EE; gender of the animals; manipulated aspects; etc.; see above). Consequently, contrasting results are reported specifically in relation to the individual components of EE (social/cognitive/physical factors; see above). Also, not univocal results are provided in relation to the different aspects of CF assessed in the tasks (intradimensional/extradimensional shift; see above). In addition, structural and biochemical correlates of EE effects on CF functions appear still not deeply and completely investigated, since an integrated analysis on the complex circuitry and molecular pathways supporting CF has not still realized.

The reported contrasting findings obtained in animals are not considerably more clarifying than the data provided by research in humans, which reports conflicting evidence about beneficial effects of enhanced experience on executive functions, when evaluated in different domains and pathological conditions (Darby et al., 2017; Hindle et al., 2017; MacPherson et al., 2019). At the current status of investigation, animal studies seem not significantly take advantage of the superior chance of variable and measure control in respect to the human studies. Thus, while a stable indication on the specific beneficial effects of EE on CF can be gained from the current state of the art in animal scientific literature, a strong suggestion on the need of further studies specifically aimed to structure a clear framework on this topic is also revealed.

## Author Contributions

FG designed the project of this review, carried out the literature search and analysis, and wrote and edited the manuscript.

## Conflict of Interest Statement

The author declares that the research was conducted in the absence of any commercial or financial relationships that could be construed as a potential conflict of interest.

## References

[B1] Anacker C. Hen R. (2017). Adult hippocampal neurogenesis and cognitive flexibility—linking memory and mood. Nat. Rev. Neurosci. 18, 335–346. 10.1038/nrn.2017.45 28469276 PMC6261347

[B2] Aumann T. D. (2016). Environment and activity-dependent dopamine neurotransmitter plasticity in the adult substantia nigra. J. Chem. Neuroanat. 73, 21–32. 10.1016/j.jchemneu.2015.12.009 26718607

[B3] Baroncelli L. Braschi C. Spolidoro M. Begenisic T. Sale A. Maffei L. (2010). Nurturing brain plasticity: impact of environmental enrichment. Cell Death Differ. 17, 1092–1103. 10.1038/cdd.2009.193 20019745

[B4] Bennett D. A. Arnold S. E. Valenzuela M. J. Brayne C. Schneider J. A. (2014). Cognitive and social lifestyle: links with neuropathology and cognition in late life. Acta Neuropathol. 127, 137–150. 10.1007/s00401-013-1226-2 24356982 PMC4054865

[B5] Berg E. A. (1948). A simple objective technique for measuring flexibility in thinking. J. Gen. Psychol. 39, 15–22. 10.1080/00221309.1948.9918159 18889466

[B6] Bergami M. (2015). Experience-dependent plasticity of adult-born neuron connectivity. Commun. Integr. Biol. 8:e1038444. 10.1080/19420889.2015.1038444 26479270 PMC4594429

[B7] Bizon J. L. Foster T. C. Alexander G. E. Glisky E. L. (2012). Characterizing cognitive aging of working memory and executive function in animal models. Front. Aging Neurosci. 4:19. 10.3389/fnagi.2012.00019 22988438 PMC3439637

[B8] Blanchet S. Chikhi S. Maltais D. (2018). The benefits of physical activities on cognitive and mental health in healthy and pathological aging. Geriatr. Psychol. Neuropsychiatr. Vieil. 16, 197–205. 10.1684/pnv.2018.0734 29877188

[B9] Bleimeister I. H. Wolff M. Lam T. R. Brooks D. M. Patel R. Cheng J. P.. (2019). Environmental enrichment and amantadine confer individual but nonadditive enhancements in motor and spatial learning after controlled cortical impact injury. Brain Res. 1714, 227–233. 10.1016/j.brainres.2019.03.007 30876859 PMC6476676

[B10] Brigman J. L. Mathur P. Harvey-White J. Izquierdo A. Saksida L. M. Bussey T. J.. (2010). Pharmacological or genetic inactivation of the serotonin transporter improves reversal learning in mice. Cereb. Cortex 20, 1955–1963. 10.1093/cercor/bhp266 20032063 PMC2912649

[B11] Brigman J. L. Powell E. M. Mittleman G. Young J. W. (2012). Examining the genetic and neural components of cognitive flexibility using mice. Physiol. Behav. 107, 666–669. 10.1016/j.physbeh.2011.12.024 22234243 PMC3337351

[B12] Brockett A. T. LaMarca E. A. Gould E. (2015). Physical exercise enhances cognitive flexibility as well as astrocytic and synaptic markers in the medial prefrontal cortex. PLoS One 10:e0124859. 10.1371/journal.pone.0124859 25938418 PMC4418599

[B13] Buttelmann F. Karbach J. (2017). Development and plasticity of cognitive flexibility in early and middle childhood. Front. Psychol. 8:1040. 10.3389/fpsyg.2017.01040 28676784 PMC5476931

[B14] Cabral J. C. Veleda G. W. Mazzoleni M. Colares E. P. Neiva-Silva L. Neves V. T. (2016). Stress and cognitive reserve as independent factors of neuropsychological performance in healthy elderly. Cien. Saude Colet. 21, 3499–3508. 10.1590/1413-812320152111.17452015 27828583

[B15] Caruso A. Nicoletti F. Mango D. Saidi A. Orlando R. Scaccianoce S. (2018). Stress as risk factor for Alzheimer’s disease. Pharmacol. Res. 132, 130–134. 10.1016/j.phrs.2018.04.017 29689315

[B16] Chakrabarti L. Scafidi J. Gallo V. Haydar T. F. (2011). Environmental enrichment rescues postnatal neurogenesis defect in the male and female Ts65Dn mouse model of Down syndrome. Dev. Neurosci. 33, 428–441. 10.1159/000329423 21865665 PMC3254041

[B17] Chan D. Shafto M. Kievit R. Matthews F. Spink M. Valenzuela M.. (2018). Lifestyle activities in mid-life contribute to cognitive reserve in late-life, independent of education, occupation, and late-life activities. Neurobiol. Aging 70, 180–183. 10.1016/j.neurobiolaging.2018.06.012 30025291 PMC6805221

[B18] Christie G. J. Hamilton T. Manor B. D. Farb N. A. S. Farzan F. Sixsmith A.. (2017). Do lifestyle activities protect against cognitive decline in aging? A review. Front. Aging Neurosci. 9:381. 10.3389/fnagi.2017.00381 29209201 PMC5701915

[B19] Chudasama Y. (2011). Animal models of prefrontal-executive function. Behav. Neurosci. 125, 327–343. 10.1037/a0023766 21639603

[B20] Clare L. Wu Y. T. Teale J. C. MacLeod C. Matthews F. Brayne C.. (2017). Potentially modifiable lifestyle factors, cognitive reserve and cognitive function in later life: a cross-sectional study. PLoS Med. 14:e1002259. 10.1371/journal.pmed.1002259 28323829 PMC5360216

[B21] Cortese G. P. Olin A. O’Riordan K. Hullinger R. Burger C. (2018). Environmental enrichment improves hippocampal function in aged rats by enhancing learning and memory, LTP, and mGluR5-Homer1c activity. Neurobiol. Aging 63, 1–11. 10.1016/j.neurobiolaging.2017.11.004 29207276 PMC5801151

[B22] Curtin P. C. Farrar A. M. Oakeshott S. Sutphen J. Berger J. Mazzella M.. (2016). Cognitive training at a young age attenuates deficits in the zQ175 mouse model of HD. Front. Behav. Neurosci. 9:361. 10.3389/fnbeh.2015.00361 26793080 PMC4707270

[B23] Dalley J. W. Cardinal R. N. Robbins T. W. (2004). Prefrontal executive and cognitive functions in rodents: neural and neurochemical substrates. Neurosci. Biobehav. Rev. 28, 771–784. 10.1016/j.neubiorev.2004.09.006 15555683

[B24] Darby R. R. Brickhouse M. Wolk D. A. Dickerson B. C. Alzheimer’s Disease Neuroimaging Initiative. (2017). Effects of cognitive reserve depend on executive and semantic demands of the task. J. Neurol. Neurosurg. Psychiatry 88, 794–802. 10.1136/jnnp-2017-315719 28630377 PMC5963955

[B25] De Bartolo P. Leggio M. G. Mandolesi L. Foti F. Gelfo F. Ferlazzo F.. (2008). Environmental enrichment mitigates the effects of basal forebrain lesions on cognitive flexibility. Neuroscience 154, 444–453. 10.1016/j.neuroscience.2008.03.069 18472349

[B26] De Bartolo P. Mandolesi L. Federico F. Foti F. Cutuli D. Gelfo F.. (2009). Cerebellar involvement in cognitive flexibility. Neurobiol. Learn. Mem. 92, 310–317. 10.1016/j.nlm.2009.03.008 19362159

[B27] Diamond M. C. Law F. Rhodes H. Lindner B. Rosenzweig M. R. Krech D.. (1966). Increases in cortical depth and glia numbers in rats subjected to enriched environment. J. Comp. Neurol. 128, 117–126. 10.1002/cne.901280110 4165855

[B28] Dickson P. E. Cairns J. Goldowitz D. Mittleman G. (2017). Cerebellar contribution to higher and lower order rule learning and cognitive flexibility in mice. Neuroscience 345, 99–109. 10.1016/j.neuroscience.2016.03.040 27012612 PMC5031514

[B29] Dickson P. E. Rogers T. D. Del Mar N. Martin L. A. Heck D. Blaha C. D.. (2010). Behavioral flexibility in a mouse model of developmental cerebellar Purkinje cell loss. Neurobiol. Learn. Mem. 94, 220–228. 10.1016/j.nlm.2010.05.010 20566377 PMC2922405

[B30] Dolivo V. Taborsky M. (2017). Environmental enrichment of young adult rats (*Rattus norvegicus*) in different sensory modalities has long-lasting effects on their ability to learn *via* specific sensory channels. J. Comp. Psychol. 131, 79–88. 10.1037/com0000063 28277718

[B31] Evans I. E. M. Llewellyn D. J. Matthews F. E. Woods R. T. Brayne C. Clare L.. (2018). Social isolation, cognitive reserve, and cognition in healthy older people. PLoS One 13:e0201008. 10.1371/journal.pone.0201008 30118489 PMC6097646

[B32] Foti F. Laricchiuta D. Cutuli D. De Bartolo P. Gelfo F. Angelucci F.. (2011). Exposure to an enriched environment accelerates recovery from cerebellar lesion. Cerebellum 10, 104–119. 10.1007/s12311-010-0236-z 21113697

[B33] Freund J. Brandmaier A. M. Lewejohann L. Kirste I. Kritzler M. Krüger A.. (2013). Emergence of individuality in genetically identical mice. Science 340, 756–759. 10.1126/science.1235294 23661762

[B34] Gelfo F. Cutuli D. Foti F. Laricchiuta D. De Bartolo P. Caltagirone C.. (2011). Enriched environment improves motor function and increases neurotrophins in hemicerebellar lesioned rats. Neurorehabil. Neural Repair 25, 243–252. 10.1177/1545968310380926 20966156

[B35] Gelfo F. De Bartolo P. Giovine A. Petrosini L. Leggio M. G. (2009). Layer and regional effects of environmental enrichment on the pyramidal neuron morphology of the rat. Neurobiol. Learn. Mem. 91, 353–365. 10.1016/j.nlm.2009.01.010 19340947

[B36] Gelfo F. Florenzano F. Foti F. Burello L. Petrosini L. De Bartolo P. (2016). Lesion-induced and activity-dependent structural plasticity of Purkinje cell dendritic spines in cerebellar vermis and hemisphere. Brain Struct. Funct. 221, 3405–3426. 10.1007/s00429-015-1109-5 26420278

[B37] Gelfo F. Mandolesi L. Serra L. Sorrentino G. Caltagirone C. (2018). The neuroprotective effects of experience on cognitive functions: evidence from animal studies on the neurobiological bases of brain reserve. Neuroscience 370, 218–235. 10.1016/j.neuroscience.2017.07.065 28827089

[B38] Girotti M. Adler S. M. Bulin S. E. Fucich E. A. Paredes D. Morilak D. A. (2018). Prefrontal cortex executive processes affected by stress in health and disease. Prog. Neuropsychopharmacol. Biol. Psychiatry 85, 161–179. 10.1016/j.pnpbp.2017.07.004 28690203 PMC5756532

[B39] Gonçalves L. V. Herlinger A. L. Ferreira T. A. A. Coitinho J. B. Pires R. G. W. Martins-Silva C. (2018). Environmental enrichment cognitive neuroprotection in an experimental model of cerebral ischemia: biochemical and molecular aspects. Behav. Brain Res. 348, 171–183. 10.1016/j.bbr.2018.04.023 29684474

[B40] Griesbach G. S. Hovda D. A. (2015). Cellular and molecular neuronal plasticity. Handb. Clin. Neurol. 128, 681–690. 10.1016/B978-0-444-63521-1.00042-X 25701914

[B41] Grønkjær M. Osler M. Flensborg-Madsen T. Sørensen H. J. Mortensen E. L. (2019). Associations between education and age-related cognitive changes from early adulthood to late midlife. Psychol. Aging 34, 177–186. 10.1037/pag0000332 30829528

[B42] Gulyaeva N. V. (2017). Molecular mechanisms of neuroplasticity: an expanding universe. Biochemistry Mosc. 82, 237–242. 10.1134/s0006297917030014 28320264

[B43] Hannan A. J. (2014). Environmental enrichment and brain repair: harnessing the therapeutic effects of cognitive stimulation and physical activity to enhance experience-dependent plasticity. Neuropathol. Appl. Neurobiol. 40, 13–25. 10.1111/nan.12102 24354721

[B44] Harrison D. J. Busse M. Openshaw R. Rosser A. E. Dunnett S. B. Brooks S. P. (2013). Exercise attenuates neuropathology and has greater benefit on cognitive than motor deficits in the R6/1 Huntington’s disease mouse model. Exp. Neurol. 248, 457–469. 10.1016/j.expneurol.2013.07.014 23911978

[B45] He C. Tsipis C. P. LaManna J. C. Xu K. (2017). Environmental enrichment induces increased cerebral capillary density and improved cognitive function in mice. Adv. Exp. Med. Biol. 977, 175–181. 10.1007/978-3-319-55231-6_24 28685443

[B46] Hebb D. O. (1949). The Organization of Behavior: A Neuropsychological Theory. New York, NY: Wiley and Sons.

[B47] Heisler J. M. Morales J. Donegan J. J. Jett J. D. Redus L. O’Connor J. C. (2015). The attentional set shifting task: a measure of cognitive flexibility in mice. J. Vis. Exp. 96:e51944. 10.3791/51944 25741905 PMC4354620

[B48] Hindle J. V. Martin-Forbes P. A. Martyr A. Bastable A. J. M. Pye K. L. Mueller Gathercole V. C.. (2017). The effects of lifelong cognitive lifestyle on executive function in older people with Parkinson’s disease. Int. J. Geriatr. Psychiatry 32, e157–e165. 10.1002/gps.4677 28170111

[B49] Hirase H. Shinohara Y. (2014). Transformation of cortical and hippocampal neural circuit by environmental enrichment. Neuroscience 280, 282–298. 10.1016/j.neuroscience.2014.09.031 25242640

[B50] Izquierdo A. Belcher A. M. Scott L. Cazares V. A. Chen J. O’Dell S. J.. (2010). Reversal-specific learning impairments after a binge regimen of methamphetamine in rats: possible involvement of striatal dopamine. Neuropsychopharmacology 35, 505–514. 10.1038/npp.2009.155 19794407 PMC2795129

[B51] Izquierdo A. Brigman J. L. Radke A. K. Rudebeck P. H. Holmes A. (2017). The neural basis of reversal learning: an updated perspective. Neuroscience 345, 12–26. 10.1016/j.neuroscience.2016.03.021 26979052 PMC5018909

[B52] Izquierdo A. Wiedholz L. M. Millstein R. A. Yang R. J. Bussey T. J. Saksida L. M.. (2006). Genetic and dopaminergic modulation of reversal learning in a touchscreen-based operant procedure for mice. Behav. Brain Res. 171, 181–188. 10.1016/j.bbr.2006.03.029 16713639

[B53] Jett J. D. Bulin S. E. Hatherall L. C. McCartney C. M. Morilak D. A. (2017). Deficits in cognitive flexibility induced by chronic unpredictable stress are associated with impaired glutamate neurotransmission in the rat medial prefrontal cortex. Neuroscience 346, 284–297. 10.1016/j.neuroscience.2017.01.017 28131625 PMC5344040

[B54] Jin X. Li T. Zhang L. Ma J. Yu L. Li C.. (2017). Environmental enrichment improves spatial learning and memory in vascular dementia rats with activation of Wnt/β-catenin signal pathway. Med. Sci. Monit. 23, 207–215. 10.12659/msm.902728 28082734 PMC5253348

[B55] Jurgens H. A. Johnson R. W. (2012). Environmental enrichment attenuates hippocampal neuroinflammation and improves cognitive function during influenza infection. Brain Behav. Immun. 26, 1006–1016. 10.1016/j.bbi.2012.05.015 22687335 PMC3454448

[B56] Kempermann G. (2019). Environmental enrichment, new neurons and the neurobiology of individuality. Nat. Rev. Neurosci. 20, 235–245. 10.1038/s41583-019-0120-x 30723309

[B57] Kesner R. P. Churchwell J. C. (2011). An analysis of rat prefrontal cortex in mediating executive function. Neurobiol. Learn. Mem. 96, 417–431. 10.1016/j.nlm.2011.07.002 21855643

[B58] Klanker M. Feenstra M. Denys D. (2013). Dopaminergic control of cognitive flexibility in humans and animals. Front. Neurosci. 7:201. 10.3389/fnins.2013.00201 24204329 PMC3817373

[B59] Kondo M. (2017). Molecular mechanisms of experience-dependent structural and functional plasticity in the brain. Anat. Sci. Int. 92, 1–17. 10.1007/s12565-016-0358-6 27484433

[B60] Kuiper J. S. Zuidersma M. Zuidema S. U. Burgerhof J. G. Stolk R. P. Oude Voshaar R. C.. (2016). Social relationships and cognitive decline: a systematic review and meta-analysis of longitudinal cohort studies. Int. J. Epidemiol. 45, 1169–1206. 10.1093/ije/dyw089 27272181

[B61] Lange F. Seer C. Kopp B. (2017). Cognitive flexibility in neurological disorders: cognitive components and event-related potentials. Neurosci. Biobehav. Rev. 83, 496–507. 10.1016/j.neubiorev.2017.09.011 28903059

[B62] Leggio M. G. Mandolesi L. Federico F. Spirito F. Ricci B. Gelfo F.. (2005). Environmental enrichment promotes improved spatial abilities and enhanced dendritic growth in the rat. Behav. Brain Res. 163, 78–90. 10.1016/j.bbr.2005.04.009 15913801

[B63] Li J. Park E. Zhong L. R. Chen L. (2019). Homeostatic synaptic plasticity as a metaplasticity mechanism - a molecular and cellular perspective. Curr. Opin. Neurobiol. 54, 44–53. 10.1016/j.conb.2018.08.010 30212714 PMC6361678

[B64] Lista I. Sorrentino G. (2010). Biological mechanisms of physical activity in preventing cognitive decline. Cell. Mol. Neurobiol. 30, 493–503. 10.1007/s10571-009-9488-x 20041290 PMC11498799

[B65] Logue S. F. Gould T. J. (2014). The neural and genetic basis of executive function: attention, cognitive flexibility, and response inhibition. Pharmacol. Biochem. Behav. 123, 45–54. 10.1016/j.pbb.2013.08.007 23978501 PMC3933483

[B66] MacPherson S. E. Gillebert C. R. Robinson G. A. Vallesi A. (2019). Editorial: intra- and inter-individual variability of executive functions: determinant and modulating factors in healthy and pathological conditions. Front. Psychol. 10:432. 10.3389/fpsyg.2019.00432 30906272 PMC6418029

[B67] Mandolesi L. Gelfo F. Serra L. Montuori S. Polverino A. Curcio G.. (2017). Environmental factors promoting neural plasticity: insights from animal and human studies. Neural Plast. 2017:7219461. 10.1155/2017/7219461 28740740 PMC5504954

[B68] Mo C. Renoir T. Hannan A. J. (2016). What’s wrong with my mouse cage? Methodological considerations for modeling lifestyle factors and gene-environment interactions in mice. J. Neurosci. Methods 265, 99–108. 10.1016/j.jneumeth.2015.08.008 26279343

[B69] Mosaferi B. Babri S. Mohaddes G. Khamnei S. Mesgari M. (2015). Post-weaning environmental enrichment improves BDNF response of adult male rats. Int. J. Dev. Neurosci. 46, 108–114. 10.1016/j.ijdevneu.2015.07.008 26291061

[B70] Nilsson S. R. Alsiö J. Somerville E. M. Clifton P. G. (2015). The rat’s not for turning: dissociating the psychological components of cognitive inflexibility. Neurosci. Biobehav. Rev. 56, 1–14. 10.1016/j.neubiorev.2015.06.015 26112128 PMC4726702

[B71] Nithianantharajah J. Hannan A. J. (2006). Enriched environments, experience-dependent plasticity and disorders of the nervous system. Nat. Rev. Neurosci. 7, 697–709. 10.1038/nrn1970 16924259

[B72] Nithianantharajah J. Hannan A. J. (2009). The neurobiology of brain and cognitive reserve: mental and physical activity as modulators of brain disorders. Prog. Neurobiol. 89, 369–382. 10.1016/j.pneurobio.2009.10.001 19819293

[B73] Novkovic T. Mittmann T. Manahan-Vaughan D. (2015). BDNF contributes to the facilitation of hippocampal synaptic plasticity and learning enabled by environmental enrichment. Hippocampus 25, 1–15. 10.1002/hipo.22342 25112659

[B74] Pang T. Y. Hannan A. J. (2013). Enhancement of cognitive function in models of brain disease through environmental enrichment and physical activity. Neuropharmacology 64, 515–528. 10.1016/j.neuropharm.2012.06.029 22766390

[B75] Perneczky R. Kempermann G. Korczyn A. D. Matthews F. E. Ikram M. A. Scarmeas N.. (2019). Translational research on reserve against neurodegenerative disease: consensus report of the International Conference on Cognitive Reserve in the Dementias and the Alzheimer’s Association Reserve, Resilience and Protective Factors Professional Interest Area working groups. BMC Med. 17:47. 10.1186/s12916-019-1283-z 30808345 PMC6391801

[B76] Petrosini L. De Bartolo P. Foti F. Gelfo F. Cutuli D. Leggio M. G.. (2009). On whether the environmental enrichment may provide cognitive and brain reserves. Brain Res. Rev. 61, 221–239. 10.1016/j.brainresrev.2009.07.002 19631687

[B77] Pettigrew C. Shao Y. Zhu Y. Grega M. Brichko R. Wang M. C.. (2019). Self-reported lifestyle activities in relation to longitudinal cognitive trajectories. Alzheimer Dis. Assoc. Disord. 33, 21–28. 10.1097/wad.0000000000000281 30376509 PMC6389389

[B78] Pfeffer A. Munder T. Schreyer S. Klein C. Rasińska J. Winter Y.. (2018). Behavioral and psychological symptoms of dementia (BPSD) and impaired cognition reflect unsuccessful neuronal compensation in the pre-plaque stage and serve as early markers for Alzheimer’s disease in the APP23 mouse model. Behav. Brain Res. 347, 300–313. 10.1016/j.bbr.2018.03.030 29572105

[B79] Phillips C. (2017). Lifestyle modulators of neuroplasticity: how physical activity, mental engagement, and diet promote cognitive health during aging. Neural Plast. 2017:3589271. 10.1155/2017/3589271 28695017 PMC5485368

[B80] Prado V. F. Janickova H. Al-Onaizi M. A. Prado M. A. (2017). Cholinergic circuits in cognitive flexibility. Neuroscience 345, 130–141. 10.1016/j.neuroscience.2016.09.013 27641830

[B81] Pudas S. Rönnlund M. (in press). School performance and educational attainment as early-life predictors of age-related memory decline: protective influences in later-born cohorts. J. Gerontol. B. Psychol. Sci. Soc. Sci. [Epub ahead of print]. 10.1093/geronb/gby137 30445430

[B82] Rosenzweig M. R. Bennett E. L. (1996). Psychobiology of plasticity: effects of training and experience on brain and behavior. Behav. Brain Res. 78, 57–65. 10.1016/0166-4328(95)00216-2 8793038

[B83] Rossi Dare L. Garcia A. Alves N. Ventura Dias D. de Souza M. A. Mello-Carpes P. B. (2019). Physical and cognitive training are able to prevent recognition memory deficits related to amyloid beta neurotoxicity. Behav. Brain Res. 365, 190–197. 10.1016/j.bbr.2019.03.007 30844418

[B84] Rountree-Harrison D. Burton T. J. Leamey C. A. Sawatari A. (2018). Environmental enrichment expedites acquisition and improves flexibility on a temporal sequencing task in mice. Front. Behav. Neurosci. 12:51. 10.3389/fnbeh.2018.00051 29599712 PMC5862792

[B85] Sakalem M. E. Seidenbecher T. Zhang M. Saffari R. Kravchenko M. Wördemann S.. (2017). Environmental enrichment and physical exercise revert behavioral and electrophysiological impairments caused by reduced adult neurogenesis. Hippocampus 27, 36–51. 10.1002/hipo.22669 27701786

[B86] Saland S. K. Rodefer J. S. (2011). Environmental enrichment ameliorates phencyclidine-induced cognitive deficits. Pharmacol. Biochem. Behav. 98, 455–461. 10.1016/j.pbb.2011.02.014 21334364

[B87] Sale A. Berardi N. Maffei L. (2014). Environment and brain plasticity: towards an endogenous pharmacotherapy. Physiol. Rev. 94, 189–234. 10.1152/physrev.00036.2012 24382886

[B88] Sampedro-Piquero P. Begega A. (2017). Environmental enrichment as a positive behavioral intervention across the lifespan. Curr. Neuropharmacol. 15, 459–470. 10.2174/1570159x14666160325115909 27012955 PMC5543669

[B89] Sampedro-Piquero P. Zancada-Menendez C. Begega A. (2015). Housing condition-related changes involved in reversal learning and its c-Fos associated activity in the prefrontal cortex. Neuroscience 307, 14–25. 10.1016/j.neuroscience.2015.08.038 26314630

[B90] Schrijver N. C. Pallier P. N. Brown V. J. Würbel H. (2004). Double dissociation of social and environmental stimulation on spatial learning and reversal learning in rats. Behav. Brain Res. 152, 307–314. 10.1016/j.bbr.2003.10.016 15196798

[B91] Serra L. Gelfo F. (2019). What good is the reserve? A translational perspective for the managing of cognitive decline. Neural Regen. Res. 14, 1219–1220. 10.4103/1673-5374.251328 30804252 PMC6425844

[B92] Serra L. Gelfo F. Petrosini L. Di Domenico C. Bozzali M. Caltagirone C. (2018). Rethinking the reserve with a translational approach: novel ideas on the construct and the interventions. J. Alzheimers Dis. 65, 1065–1078. 10.3233/jad-180609 30149458

[B93] Shipman M. L. Green J. T. (in press). Cerebellum and cognition: does the rodent cerebellum participate in cognitive functions? Neurobiol. Learn. Mem. [Epub ahead of print]. 10.1016/j.nlm.2019.02.006 30771461

[B94] Simpson J. Kelly J. P. (2011). The impact of environmental enrichment in laboratory rats—behavioural and neurochemical aspects. Behav. Brain Res. 222, 246–264. 10.1016/j.bbr.2011.04.002 21504762

[B95] Stern Y. (2002). What is cognitive reserve? Theory and research application of the reserve concept. J. Int. Neuropsychol. Soc. 8, 448–460. 10.1017/s1355617702813248 11939702

[B96] Stern Y. Arenaza-Urquijo E. M. Bartrés-Faz D. Belleville S. Cantilon M. Chetelat G.. (2018). Whitepaper: defining and investigating cognitive reserve, brain reserve, and brain maintenance. Alzheimers Dement. [Epub ahead of print]. 10.1016/j.jalz.2018.07.219 30222945 PMC6417987

[B97] Tait D. S. Chase E. A. Brown V. J. (2014). Attentional set-shifting in rodents: a review of behavioural methods and pharmacological results. Curr. Pharm. Des. 20, 5046–5059. 10.2174/1381612819666131216115802 24345263

[B98] Talpos J. Shoaib M. (2015). Executive function. Handb. Exp. Pharmacol. 228, 191–213. 10.1007/978-3-319-16522-6_6 25977083

[B99] Toth L. A. Kregel K. Leon L. Musch T. I. (2011). Environmental enrichment of laboratory rodents: the answer depends on the question. Comp. Med. 61, 314–321. 22330246 PMC3155397

[B100] Whitehouse C. M. Curry-Pochy L. S. Shafer R. Rudy J. Lewis M. H. (2017). Reversal learning in C58 mice: modeling higher order repetitive behavior. Behav. Brain Res. 332, 372–378. 10.1016/j.bbr.2017.06.014 28624316 PMC5553316

[B101] Yates L. A. Ziser S. Spector A. Orrell M. (2016). Cognitive leisure activities and future risk of cognitive impairment and dementia: systematic review and meta-analysis. Int. Psychogeriatr. 28, 1791–1806. 10.1017/s1041610216001137 27502691

[B102] Zeleznikow-Johnston A. Burrows E. L. Renoir T. Hannan A. J. (2017). Environmental enrichment enhances cognitive flexibility in C57BL/6 mice on a touchscreen reversal learning task. Neuropharmacology 117, 219–226. 10.1016/j.neuropharm.2017.02.009 28196627

